# Airway Ultrasound: A Narrative Review of Present Use and Future Applications in Anesthesia

**DOI:** 10.3390/healthcare13131502

**Published:** 2025-06-24

**Authors:** Efrain Riveros-Perez, Bibiana Avella-Molano, Alexander Rocuts

**Affiliations:** Department of Anesthesiology and Perioperative Management, Medical College of Georgia, Augusta University, Augusta, GA 30912, USA

**Keywords:** point-of-care ultrasound, airway management, difficult airway

## Abstract

**Introduction**: Airway management remains a high-risk intervention in a subset of patients, with traditional predictors like the Mallampati score demonstrating poor sensitivity and specificity. Point-of-care ultrasound (POCUS) has emerged as a transformative tool, offering real-time, objective assessment of airway anatomy to improve safety and outcomes. **Methods**: A narrative approach was conducted to evaluate the literature on airway ultrasound, incorporating clinical metrics and procedural applications. **Results**: Ultrasound has demonstrated utility in pre-intubation risk stratification using quantitative measures such as skin-to-epiglottis distance (>2.75 cm) and hyomental distance ratio (<1.2), which outperform traditional exams, especially in obese patients. Procedural uses include endotracheal tube confirmation with 98.9% sensitivity and enhanced success rates in emergent cricothyroidotomy—from 50% to nearly 100%—in patients with difficult anatomy. Dynamic applications like assessing laryngeal edema via parapharyngeal thickness offer advantages over traditional cuff leak tests. Technical considerations such as optimal probe selection, patient positioning, and interpretation of key anatomical landmarks are also discussed. **Conclusions**: Airway ultrasound is poised to become a standard tool in perioperative and critical care settings. The review concludes by emphasizing POCUS as an indispensable adjunct for modern airway management.

## 1. Introduction

Airway management is a cornerstone of patient care across emergency rooms, intensive care units, and operating rooms (ORs), with approximately 15 million intubations performed annually in the ORs and 650,000 in other hospital settings in the United States [1]. Despite its routine nature, airway management carries significant risks: 5–8% of tracheal intubations are difficult, and 0.05–0.35% are impossible [2]. Even more alarming, 0.0019–0.04% of cases progress to a “cannot intubate, cannot ventilate” scenario [3].

Current clinical predictors of difficult airways, such as the Mallampati score or Wilson risk assessment, suffer from poor sensitivity and specificity, with AUROCs often falling below acceptable diagnostic thresholds [4,5]. For example, the mouth opening test has a sensitivity of just 0.22, while even the best-performing predictors (e.g., the lip bite test at 0.67 sensitivity) still miss a third of high-risk cases [4]. False negatives are especially dangerous, leaving clinicians unprepared for unexpected challenges. Some experts have even questioned whether traditional screening tools offer any meaningful clinical benefit [6].

These limitations underscore the critical need for more objective, precise, and clinically practical tools to predict difficult airways. Point-of-care ultrasound (POCUS) provides a promising alternative to traditional airway assessment tools by offering real-time, objective measures and direct visualization of airway anatomy. A growing body of evidence demonstrates strong correlations between ultrasound-measured airway parameters and laryngoscopic view grades [7,8,9], positioning POCUS as a valuable adjunct in airway evaluation. This review aims to synthesize current literature on airway ultrasound for difficult airway prediction. It further seeks to evaluate the diagnostic accuracy, practical applications, and clinical impact of airway ultrasound in the perioperative setting.

## 2. Materials & Methods

A literature search was conducted using electronic databases including PubMed, MEDLINE, and Google Scholar, employing keywords such as “airway ultrasound”, “ultrasound in anesthesia”, “airway management”, “airway evaluation”, and “difficult airway”. Articles were screened by title and abstract, followed by full-text review for eligibility. Studies published from January 2000 onward were included if they were peer-reviewed original studies, review articles, guidelines, or case reports focused on the application of ultrasound in airway evaluation. Publications not available in English and those unrelated to airway management in anesthesia were excluded. The authors also incorporated their clinical experience in airway ultrasound to guide the selection and interpretation of relevant content. While no formal systematic review protocol was used, the structure and reporting of this narrative review were guided by the PRISMA-Scoping Review checklist where appropriate. Findings were organized thematically to emphasize clinical applications, techniques, limitations, and potential future directions. As this review does not involve human subjects, institutional review board (IRB) approval was not obtained.

## 3. Results

### 3.1. Airway Ultrasound: Principles and Techniques

Ultrasound imaging uses high-frequency sound waves (1–20 MHz) to generate real-time visualizations of anatomical structures. Piezoelectric crystals in the transducer convert electrical energy into sound waves and back into electrical energy as echoes return from tissue interfaces. This electrical energy is then processed into 2D or 3D images [10]. The degree of reflection of waves at tissue interfaces is determined by the acoustic impedance of the medium, a property primarily determined by tissue density [11]. For instance, dense structures such as bone exhibit high acoustic impedance, resulting in strong reflection and hyperechoic (bright) appearance on ultrasound. In contrast, air has low acoustic impedance and attenuates most ultrasound waves, generating an anechoic (dark) imaging [12]. These acoustic properties, when applied to airway evaluation, allow the distinction of soft tissues, cartilage, bone, and conductive airways. In this way, ultrasound provides a dynamic airway assessment that aids in the identification of a difficult airway [13].

Transducer selection is crucial for optimal airway imaging. Linear transducers have a high frequency (typically 7–15 MHz) that provides excellent image resolution at the expense of limited tissue penetration, making it ideal to evaluate superficial structures such as the cricothyroid membrane, vocal cords, and epiglottis [14]. In contrast, the curvilinear transducer, with a lower frequency (2–5 MHz), offers deeper penetration and a wider field of view, making it useful for evaluating the base of the tongue or the hyomental distance [14].

**Figure 1:**
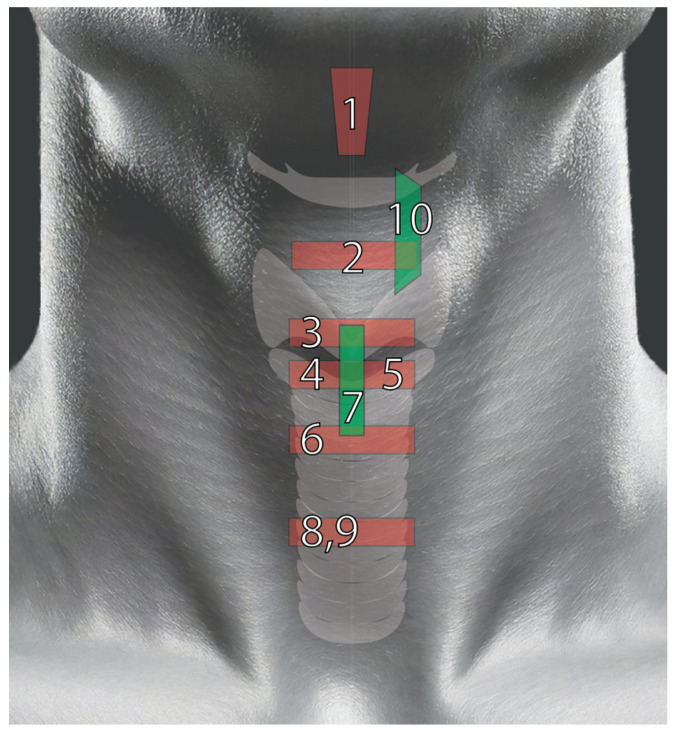
Airway ultrasound probe positions. The red boxes represent the approximate location of the footprint of the probe on the surface of the skin and its orientation (longitudinal or transverse). All views are performed with the use of a high-frequency linear probe, with the exception of the suprahyoid view. 1, Suprahyoid view, performed with a curved linear probe; 2, Thyrohyoid view; 3, Thyroid view; 4, Transverse cricothyroid membrane view; 5, Suprasternal view of the cricoid; 6, Suprasternal view of the tracheal rings; 7, Suprasternal longitudinal view; 8, Suprasternal view to identify the esophagus; 9, Suprasternal view to identify endotracheal tube; 10, Parasagital Thyrohyoid view.

Proper patient positioning is as important as correct probe selection for airway ultrasound. Ideally, the patient is positioned supine with the head in neutral position or extension. The sniffing position commonly employed during intubation aligns the oral, pharyngeal, and laryngeal axes and aids in probe manipulation by increasing the surface area [14]. Transducer positioning will depend on the targeted sonographic view, as outlined in Figure 1.

### 3.2. Sonoanatomy of the Upper Airway

**Figure 2:**
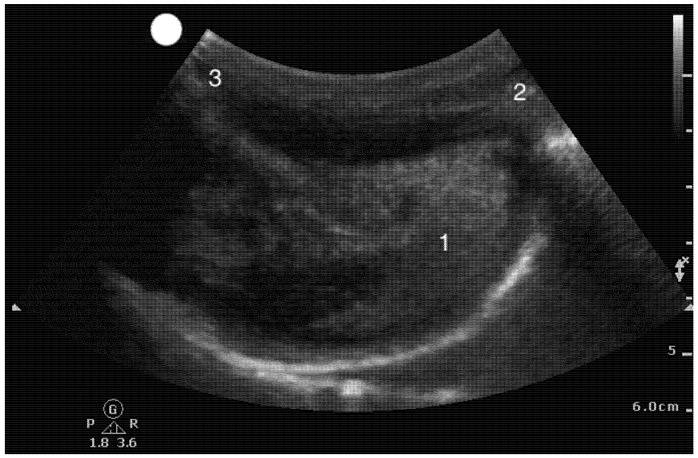
Suprahyoid view: Longitudinal view of the floor of the mouth. 1.Tongue; 2, Mentum; 3, Hyoid bone.

**Figure 3:**
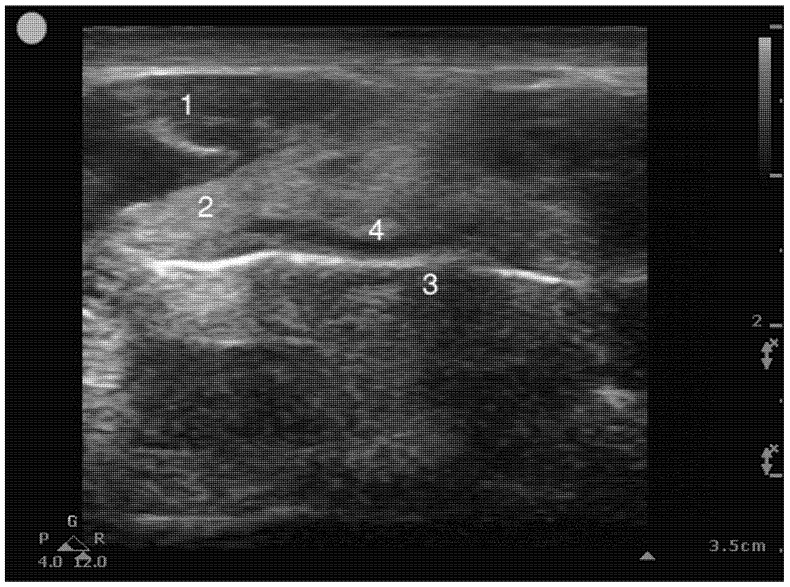
Transverse Thyrohyoid view. 1, Strap muscles; 2, Periepiglotic space; 3, Air mucosa interface; 4. The epiglottis, which appears as a hypoechoic structure.

**Figure 4:**
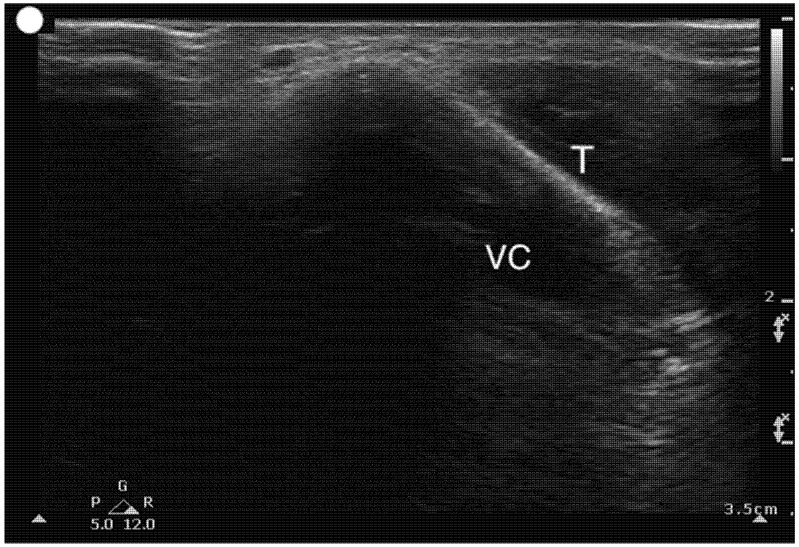
Transverse Thyroid view. T, Thyroid cartilage; VC, Vocal cord muscles.

**Figure 5:**
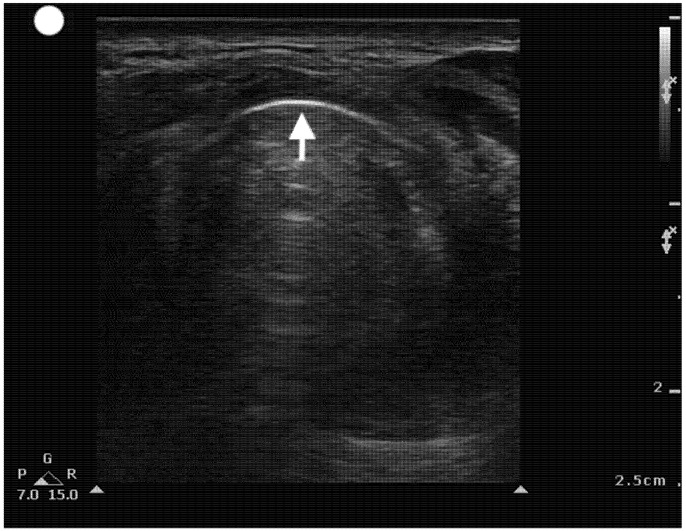
Transverse Cricothyroid view. Arrow pointing towards the crico-thyroid membrane. Reverberation artifacts are seen parallel and in the far field of view.

**Figure 6:**
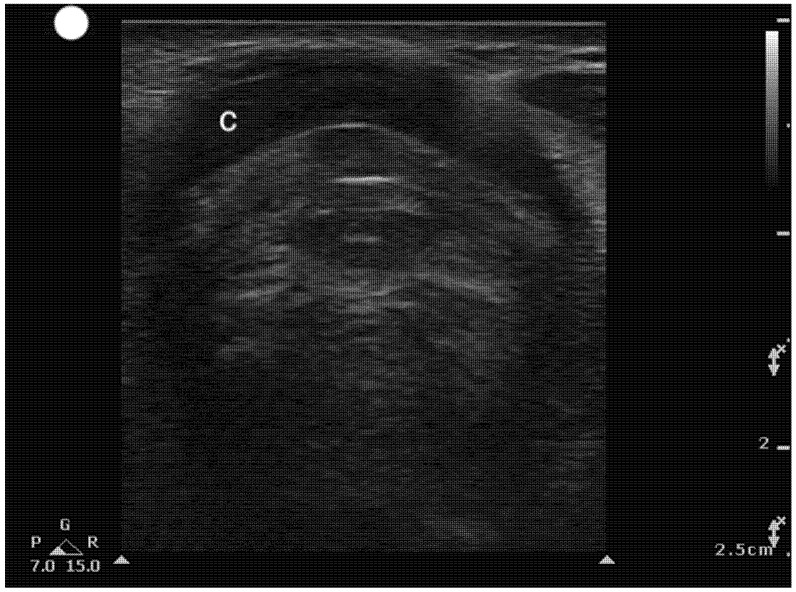
Transverse Suprasternal view at the level of the cricoid cartilage. C, Cricoid cartilage.

**Figure 7:**
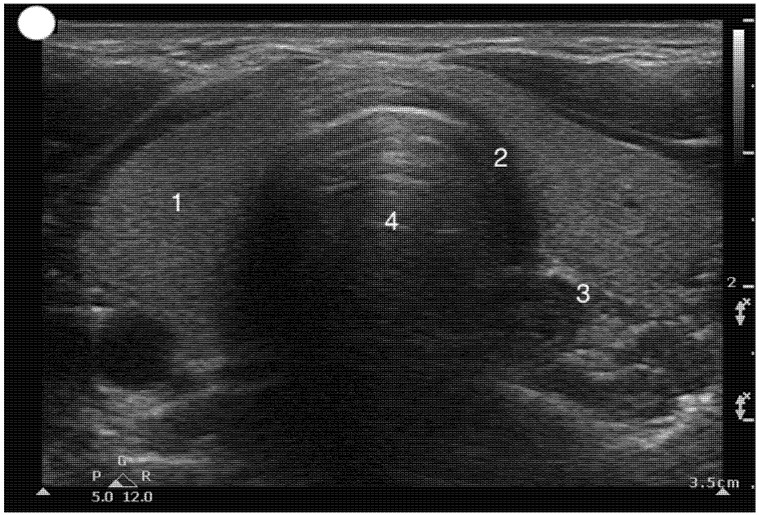
Transverse Suprasternal view at the level of the tracheal rings. 1, Left lobe of the thyroid gland connected to the right by its isthmus; 2, Tracheal ring; 3. Esophagus; 4. Trachea.

**Figure 8:**
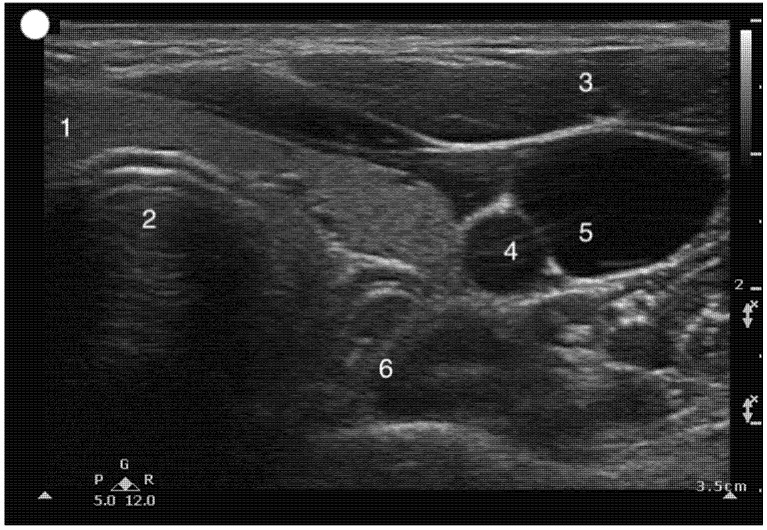
Transverse Suprasternal view to visualize the esophagus. 1, Thyroid gland; 2, Trachea; 3. Sternomastoid muscle; 4, Carotid; 5, Internal Jugular vein; 6. Esophagus.

**Figure 9:**
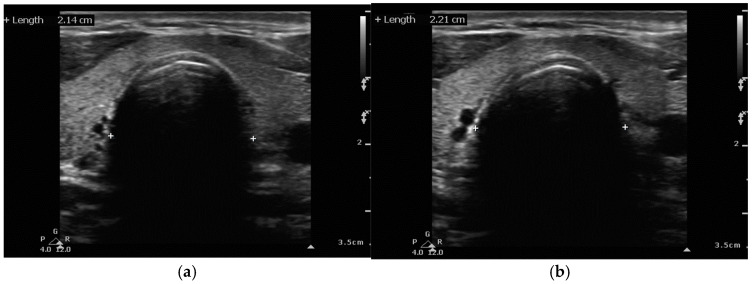
Transverse suprasternal view without (**a**) and with (**b**) endotracheal tube cuff inflated. Distances displayed are measured from approximately the same anatomical landmarks.

**Figure 10:**
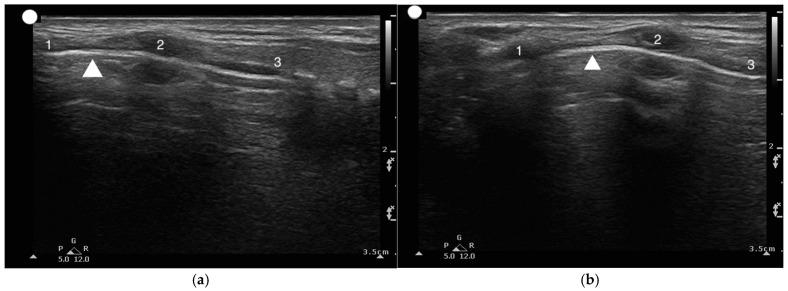
(**a**,**b**) Longitudinal view of the upper airway to visualize the String of Pearls. 1, Thyroid cartilage; 2, Cricoid cartilage; 3. Tracheal rings; The arrowhead points towards the crico-thyroid membrane.

**Figure 11:**
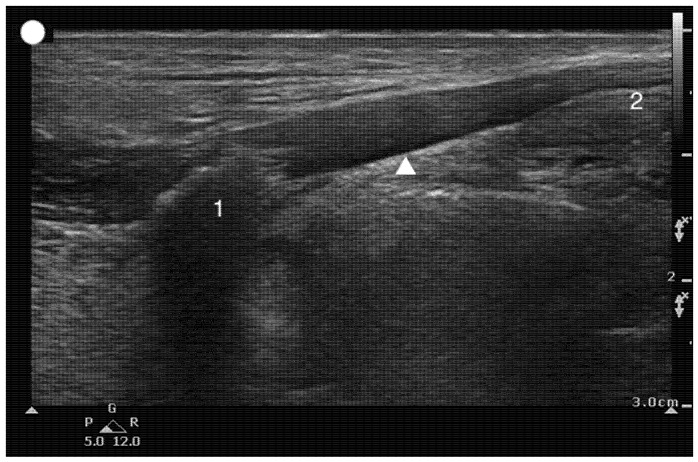
Parasagittal Thyrohyoid view. 1, Hyoid bone; 2, Thyroid cartilage; The arrowhead marks the location of the thyrohyoid membrane.

Airway sonographic evaluation employs multiple views to optimize anatomical visualization and improve diagnostic accuracy. The sagittal suprahyoid view allows for detailed evaluation of the supraepiglottic space and measures hyomental distance (Figure 2). The thyrohyoid view is used to identify the hypoechogenic epiglottis, which are described as the mouth in the “small face sign” (Figure 3). Visualization of the true vocal cords is achieved via the transverse thyroid view, where the cords appear as paired hypoechoic structures lateral to the midline (Figure 4, Video S1). The transverse cricothyroid view is used to identify the cricothyroid membrane, which is often seen with reverberation artifact in the tracheal lumen (Figure 5). The transverse suprasternal view enables assessment of the esophagus, typically located left of the trachea, and confirmation of endotracheal tube (ETT) placement by identifying a single air-mucosa interface within the trachea (Figure 6, Figure 7, Figure 8 and Figure 9). A longitudinal midline view facilitates identification of the tracheal rings and cricoid cartilage, often described as a “string of pearls,” and aids in accurate localization of the cricothyroid membrane (Figure 10, Video S2). Finally, Figure 11 shows the paramedian thyrohyoid view that provides access to the pre-epiglottic space and is useful for superior laryngeal nerve blocks to enhance safety and efficacy [15,16,17].

### 3.3. Airway Ultrasound in Specific Clinical Situations

Ultrasound serves as a valuable tool for comprehensive airway assessment, offering several key clinical applications: (1) pre-intubation evaluation of anatomical predictors of difficult airway (e.g., hyomental distance, tongue thickness); (2) real-time confirmation of endotracheal tube placement; (3) dynamic assessment of airway anatomy to identify developing edema or predict post-extubation stridor by measuring vocal cord and subglottic dimensions; and (4) guided localization of the cricothyroid membrane for emergency surgical airway access, significantly improving the success rate of cricothyroidotomy procedures.

#### 3.3.1. Assessment of Difficulty with Airway

Quantitative airway ultrasound assessment, particularly measurement of the *skin-to-epiglottis distance (DSE)*, has emerged as a reliable predictor of difficult laryngoscopy. The standardized measurement technique involves: (1) placing a high-frequency linear transducer in the transverse plane at the level of the thyroid membrane, (2) maintaining the patient in a supine position with neutral head alignment, and (3) systematically scanning from the submental region to the sternal notch until identifying the characteristic hypoechoic curvilinear structure of the epiglottis.

Meta-analysis data demonstrate that a DSE > 2.5 cm strongly correlates with Cormack-Lehane grades III/IV with high negative predictive value (95–97%) [8]. Subsequent validation by Pinto et al. established an optimized cutoff of 2.75 cm, achieving balanced test characteristics (accuracy: 74.3%; sensitivity: 64.7%; specificity: 77.1%). When integrated with the modified Mallampati score, DSE provides superior predictive capability compared to clinical assessment alone [18]. These findings extend to pediatric populations, where DSE measurement shows diagnostic value in children aged 5–8 years (AUROC: 0.76; sensitivity: 88%; *n* = 310) [19].

*Hyomental distance (HMD) and hyomental distance ratio (HMDR)* are well-validated sonographic predictors of difficult laryngoscopy. Measured in a midline sagittal view over the submental region, HMD represents the distance between the mentum of the mandible and the superior border of the hyoid bone, while HMDR is calculated as the ratio of HMD in neutral versus head-extended positions. An HMDR < 1.2 has high sensitivity (95%) for difficult intubation [20,21]. Notably, in obese patients, HMDR shows significantly greater reliability than clinical assessment alone (Pearson r = 0.14), reinforcing its utility in this high-risk population [22].

*The skin-to-vocal cord distance (SVC)* is measured via transverse ultrasound at the thyroid cartilage level, with phonation used to dynamically differentiate true vocal cords (mobile) from false cords (fixed). In obese patients, pre-tracheal tissue thickness >28 mm anterior to the vocal cords predicts difficult laryngoscopy [23]. Likewise, in the general adult population, higher SVC values have consistently demonstrated a strong association with difficult laryngoscopy and difficult intubation [9,24].

*The skin-to-hyoid bone distance (SHB)*, measured via transverse ultrasound over the hyoid body, serves as both an anatomical and quantitative predictor of airway difficulty. Direct visualization of the hyoid bone correlates with favorable Cormack-Lehane grades (I-II) and easier laryngoscopy [25], while SHB measurement demonstrates moderate diagnostic accuracy (sensitivity 68%, specificity 73%) for difficult airways [26]. A pilot study found ultrasound assessments, including SHB, superior to clinical screening tests [27], although other studies highlight its synergistic role: SHB and thyrohyoid membrane measurements emerge as independent yet strongly correlated predictors (r = 0.74) of difficult intubation [28].

Ultrasound measurement of tongue thickness has been strongly associated with difficult intubation, demonstrating excellent sensitivity (100%) and specificity (83%) for predicting difficult laryngoscopy [29]. These findings were corroborated by a large multicenter study (*n* = 2254) where tongue thickness >6.1 cm independently predicted difficult intubation (AUROC 0.78, 95% CI 0.77–0.80) [30]. Wang et al. developed a nomogram combining tongue thickness and mobility with traditional assessments, achieving exceptional discrimination (AUROC 0.93 for difficult laryngoscopy; 0.97 for difficult intubation) [31]. Similarly, a validated risk scoring system incorporating ultrasound measurements (skin-to-thyrohyoid membrane >1.60 cm [2 points], skin-to-thyroid cartilage >0.78 cm [3 points]) and male gender (2 points) demonstrated strong predictive value, with scores ≥5 associated with a 34-fold increased risk of difficult laryngoscopy (*p* = 0.001, sensitivity 91.67%, specificity 75.56%) [32]. These multimodal approaches represent significant progress in preoperative airway assessment. Table 1 summarizes all the aforementioned sonographic predictors and their diagnostic accuracy.

#### 3.3.2. Confirmation of Endotracheal Tube Position

The gold standard for confirming endotracheal tube placement is the detection of a capnogram waveform. However, in certain clinical scenarios, such as cardiac arrest (with extremely low pulmonary blood flow), severe bronchospasm, or situations requiring real-time airway assessment, capnography may be unreliable or impractical. In these cases, ultrasound emerges as a valuable alternative. Using a convex transducer via a suprasternal view, Chou et al. reported a sensitivity of 98.9% and specificity of 94.1% [33]. Further supporting its utility, a prospective study of 107 patients found near-perfect agreement (κ = 0.85) between ultrasound confirmation and capnography, with an average confirmation time of just 16.4 s [34]. A meta-analysis of 12 studies, including both adult patients and cadaveric models, reinforced these findings, demonstrating an AUROC of 0.97 for ultrasound in verifying endotracheal intubation [35]. These results underscore the clinical value of ultrasound as an effective adjunct for airway confirmation.

#### 3.3.3. Evaluation of Laryngeal Edema

Airway edema is a critical concern in patients with prolonged intubation, airway trauma, or significant intraoperative fluid shifts. While the cuff leak test (CLT) is commonly used to assess extubation readiness, its low sensitivity (66%) limits its reliability as a standalone screening tool despite high specificity [36]. Airway ultrasound offers a valuable alternative, enhancing clinical decision-making by objectively evaluating edema. For instance, measuring the width of the air column during cuff deflation can detect mild to moderate edema [37], and multiple studies support its clinical utility in improving extubation outcomes [38]. Notably, a recent study demonstrated that point-of-care ultrasound (POCUS) effectively identified postoperative increases in parapharyngeal thickness, lateral pharyngeal wall thickness, and neck circumference—changes that correlated with intraoperative fluid administration—further validating its role in detecting airway edema, particularly after procedures like robotic prostatectomy in the Trendelenburg position [39].

#### 3.3.4. Ultrasound to Aid in Emergent Cricothyroidotomy

Cricothyroidotomy is a critical rescue procedure in “cannot intubate, cannot ventilate” scenarios, yet anesthesiologists succeed in performing needle cricothyroidotomy in only ~50% of cases, compared to near 100% success rates among surgical specialists [40]. While surgical cricothyroidotomy may be more reliable than percutaneous approaches, it carries higher risks of complications. Ultrasound offers a potential solution by improving the accuracy of cricothyroid membrane identification, either statically or in real time, enhancing both the safety and efficacy of percutaneous techniques [41]. Studies in simulated emergencies show that although palpation is faster, ultrasound matches it in provider confidence and preference [42]. Moreover, in patients with obscured neck anatomy (e.g., obesity, trauma, or edema), ultrasound outperforms palpation in reliably locating the cricothyroid membrane [43], making it an indispensable tool in high-stakes airway emergencies.

#### 3.3.5. Limitations

Despite the broad advantages of airway ultrasound, it has some limitations. Its diagnostic accuracy is highly operator-dependent, with a variable learning curve among practitioners. Limited access to ultrasound equipment can also restrict opportunities for skill development, particularly in low-resource settings [14]. Patient-related factors, such as obesity or anatomical variations, may impede clear visualization and correct identification of airway structures [44]. Finally, like other predictors of difficult airway, ultrasound should not be used in isolation but rather as a complementary tool alongside clinical assessment and other diagnostic methods to enhance overall accuracy [45].

On the other hand, this article has limitations inherent to its design. As a narrative review, we did not perform a systematic search of the literature, apply strict inclusion and exclusion criteria, or evaluate the methodological quality of each study. Nevertheless, we believe that a narrative approach was the most suitable for our objective, which was to provide a comprehensive overview of the current and potential future applications of ultrasound in airway management within anesthesia. This approach allowed us to highlight key concepts, techniques, and innovations in a flexible and thorough manner, reflecting the current state of knowledge and guiding future practice and research.

## 4. Conclusions

Airway ultrasound has emerged as a transformative tool in modern anesthesia and critical care, offering objective, real-time assessment of airway anatomy to address the limitations of traditional clinical predictors. From pre-intubation risk stratification using metrics like skin-to-epiglottis distance and hyomental ratio to dynamic evaluation of laryngeal edema and confirmation of endotracheal tube placement, ultrasound enhances accuracy, safety, and clinical decision-making across diverse scenarios. Its utility in emergent cricothyroidotomy, particularly in patients with obscured anatomy, further underscores its lifesaving potential. While challenges such as operator skill and accessibility remain, the integration of ultrasound into routine practice, supported by robust evidence, promises to reduce complications, improve outcomes, and redefine standards in airway management. Future advancements, including AI-assisted imaging and portable technologies, may further solidify its role as an indispensable adjunct in perioperative and emergency care.

## Figures and Tables

**Table 1:** Sonographic Predictors of Difficult Airway and Their Diagnostic Accuracy.

Sonographic Predictor	Measurement Cut-Off	Sensitivity (%)	Specificity (%)	Additional Notes
Distance from Skin to Epiglottis (DSE)	>2.5–2.75 cm	64.7	77.1	Strong NPV; increased accuracy when combined with Mallampati (compared to clinical assessment alone)
Hyomental Distance Ratio (HMDR)	<1.2	88–95.2	60–69.2	Superior to traditional clinical predictors; especially useful in obese patients.
Skin-to-Vocal Cord Distance (SVC)	>9.4 mm	70	84	Increased SVC correlates with difficult laryngoscopy, especially in high-BMI patients.
Skin-to-Hyoid Bone Distance (SHB)	66–77 mm	68	73	
Tongue Thickness	>6.1 cm	75	83	Its high sensitivity and specificity make this measurement excellent for evaluating difficult intubation.

NPV, Negative Predictive Value; BMI, body mass index.

## Data Availability

Not applicable.

## Supplementary Materials

The following supporting information can be downloaded at: https://www.mdpi.com/article/10.3390/healthcare13131502/s1, Video S1. Transverse Thyroid view showing vocal cord movement. Video S2. Longitudinal view of the airway as the linear probe ascends from a suprasternal view to visualize the cricothyroid membrane. SOP, string of pearls. C, cricoid cartilage; T, Thyroid cartilage.

## References

[1] Turner J.S., Bucca A.W., Propst S.L., Ellender T.J., Sarmiento E.J., Menard L.M., Hunter B.R. (2020). Association of Checklist Use in Endotracheal Intubation with Clinically Important Outcomes: A Systematic Review and Meta-analysis. JAMA Netw. Open.

[2] Levitan R.M., Heitz J.W., Sweeney M., Cooper R.M. (2011). The complexities of tracheal intubation with direct laryngoscopy and alternative intubation devices. Ann. Emerg. Med..

[3] Kwon Y.S., Lee C.A., Park S., Ha S.O., Sim Y.S., Baek M.S. (2019). Incidence and outcomes of cricothyrotomy in the “cannot intubate, cannot oxygenate” situation. Medicine.

[4] Roth D., Pace N.L., Lee A., Hovhannisyan K., Warenits A.-M., Arrich J., Herkner H. (2018). Airway physical examination tests for detection of difficult airway management in apparently normal adult patients. Cochrane Database Syst. Rev..

[5] Heidegger T., Pandit J.J. (2025). Difficult Airway Management: From the Power of Prediction to the Art of Management. Anesth. Analg..

[6] Yentis S.M. (2002). Predicting difficult intubation–worthwhile exercise or pointless ritual?. Anaesthesia.

[7] Sotoodehnia M., Rafiemanesh H., Mirfazaelian H., Safaie A., Baratloo A. (2021). Ultrasonography indicators for predicting difficult intubation: A systematic review and meta-analysis. BMC Emerg. Med..

[8] Carsetti A., Sorbello M., Adrario E., Donati A., Falcetta S. (2022). Airway Ultrasound as Predictor of Difficult Direct Laryngoscopy: A Systematic Review and Meta-analysis. Anesth. Analg..

[9] Gomes S.H., Simões A.M., Nunes A.M., Pereira M.V., Teoh W.H., Costa P.S., Kristensen M.S., Teixeira P.M., Pêgo J.M. (2021). Useful Ultrasonographic Parameters to Predict Difficult Laryngoscopy and Difficult Tracheal Intubation—A Systematic Review and Meta-Analysis. Front. Med..

[10] Borowy C.S., Mukhdomi T. (2023). Sonography Physical Principles and Instrumentation. StatPearls [Internet].

[11] Bakhru R.N., Schweickert W.D. (2013). Intensive care ultrasound: I. Physics, equipment, and image quality. Ann. Am. Thorac. Soc..

[12] Sidebotham D., Merry A.F., Legget M.E., Wright I.G. (2018). Practical Perioperative Transoesophageal Echocardiography.

[13] Garg R., Gupta A. (2015). Ultrasound: A promising tool for contemporary airway management. World J. Clin. Cases.

[14] Lin J., Bellinger R., Shedd A., Wolfshohl J., Walker J., Healy J., Taylor J., Chao K., Yen Y.-H., Tzeng C.-F.T. (2023). Point-of-care ultrasound in airway evaluation and management: A comprehensive review. Diagnostics.

[15] Kadadi S., Gadkari C., Pundkar A. (2024). A Comparison of Airway Ultrasound and Clinical Parameters for Predicting Difficult Laryngoscopy in the Emergency Department: A Study Protocol. Cureus.

[16] Singh M., Chin K.J., Chan V.W., Wong D.T., Prasad G.A., Yu E. (2010). Use of sonography for airway assessment: An observational study. J. Ultrasound Med..

[17] Shan T., Tan Q., Wu D., Bao H., Ge D., Han L., Su C., Ju Y. (2024). Ultrasound-guided superior laryngeal nerve block: A randomized comparison between parasagittal and transverse approach. BMC Anesthesiol..

[18] Pinto J., Cordeiro L., Pereira C., Gama R., Fernandes H., Assunção J. (2016). Predicting difficult laryngoscopy using ultrasound measurement of distance from skin to epiglottis. J. Crit. Care.

[19] Zheng Z., Wang X., Du R., Wu Q., Chen L., Ma W. (2023). Effectiveness of ultrasonic measurement for the hyomental distance and distance from skin to epiglottis in predicting difficult laryngoscopy in children. Eur. Radiol..

[20] Kalezić N., Lakićević M., Miličić B., Stojanović M., Sabljak V., Marković D. (2016). Hyomental distance in the different head positions and hyomental distance ratio in predicting difficult intubation. Bosn. J. Basic Med Sci..

[21] Huh J., Shin H.-Y., Kim S.-H., Yoon T.-K., Kim D.-K. (2009). Diagnostic predictor of difficult laryngoscopy: The hyomental distance ratio. Anesth. Analg..

[22] Petrișor C., Trancă S., Szabo R., Simon R., Prie A., Bodolea C. (2020). Clinical versus Ultrasound Measurements of Hyomental Distance Ratio for the Prediction of Difficult Airway in Patients with and without Morbid Obesity. Diagnostics.

[23] Osman A., Sum K.M. (2016). Role of upper airway ultrasound in airway management. J. Intensiv. Care.

[24] Sotoodehnia M., Khodayar M., Jalali A., Momeni M., Safaie A., Abdollahi A. (2023). Prediction of difficult laryngoscopy/difficult intubation cases using upper airway ultrasound measurements in emergency department: A prospective observational study. BMC Emerg. Med..

[25] Petrisor C., Szabo R., Constantinescu C., Prie A., Hagau N. (2018). Ultrasound-based assessment of hyomental distances in neutral, ramped, and maximum hyperextended positions, and derived ratios, for the prediction of difficult airway in the obese population: A pilot diagnostic accuracy study. Anaesthesiol. Intensiv. Ther..

[26] Rudingwa P., Yadav N.K., Mishra S.K., Pannerselvam S. (2019). Ultrasound measurement of anterior neck soft tissue and tongue thickness to predict difficult laryngoscopy—An observational analytical study. Indian J. Anaesth..

[27] Adhikari S., Zeger W., Schmier C., Crum T., Craven A., Frrokaj I., Pang H., Shostrom V. (2011). Pilot study to determine the utility of point-of-care ultrasound in the assessment of difficult laryngoscopy. Acad. Emerg. Med..

[28] Zheng J., Wu J., Dong J., Ding Y. (2014). Role of anterior neck soft tissue quantifications by ultrasound in predicting difficult laryngoscopy. Med Sci. Monit..

[29] Furia J.S., Nadkarni M.M. (2024). Ultrasound-based assessment of tongue thickness for prediction of difficult laryngoscopy and intubation. J. Anaesthesiol. Clin. Pharmacol..

[30] Yao W., Wang B. (2017). Can tongue thickness measured by ultrasonography predict difficult tracheal intubation?. Br. J. Anaesth..

[31] Wang B., Yao W., Xue Q., Wang M., Xu J., Chen Y., Zhang Y. (2022). Nomograms for predicting difficult airway based on ultrasound assessment. BMC Anesthesiol..

[32] De Luis-Cabezón N., Ly-Liu D., Renedo-Corcostegui P., Santaolalla-Montoya F., de Maturana A.Z.-L., Herrero-Herrero J.C., Martínez-Hurtado E., De Frutos-Parra R., Bilbao-Gonzalez A., Fernandez-Vaquero M.A. (2024). A new score for airway assessment using clinical and ultrasound parameters. Front. Med..

[33] Chou H.-C., Tseng W.-P., Wang C.-H., Ma M.H.-M., Wang H.-P., Huang P.-C., Sim S.-S., Liao Y.-C., Chen S.-Y., Hsu C.-Y. (2011). Tracheal rapid ultrasound exam (T.R.U.E.) for confirming endotracheal tube placement during emergency intubation. Resuscitation.

[34] Adi O., Chuan T.W., Rishya M. (2013). A feasibility study on bedside upper airway ultrasonography compared to waveform capnography for verifying endotracheal tube location after intubation. Crit. Ultrasound J..

[35] Chou E.H., Dickman E., Tsou P.-Y., Tessaro M., Tsai Y.-M., Ma M.H.-M., Lee C.-C., Marshall J. (2015). Ultrasonography for confirmation of endotracheal tube placement: A systematic review and meta-analysis. Resuscitation.

[36] Kuriyama A., Jackson J.L., Kamei J. (2020). Performance of the cuff leak test in adults in predicting post-extubation airway complications: A systematic review and meta-analysis. Crit. Care.

[37] Pluijms W.A., van Mook W.N., Wittekamp B.H., Bergmans D.C. (2015). Postextubation laryngeal edema and stridor resulting in respiratory failure in critically ill adult patients: Updated review. Crit. Care.

[38] Schick M., Grether-Jones K. (2016). Point-of-Care Sonographic Findings in Acute Upper Airway Edema. West J. Emerg. Med..

[39] Aytac B.G., Soyal O.B. (2023). Ultrasonographic evaluation of the postoperative airway edema after robotic prostatectomy: A single center observational study. Eur. Rev. Med. Pharmacol. Sci..

[40] Cook T.M., Woodall N., Harper J., Benger J. (2011). Fourth National Audit Project. Major complications of airway management in the UK: Results of the Fourth National Audit Project of the Royal College of Anaesthetists and the Difficult Airway Society. Part 2: Intensive care and emergency departments. Br. J. Anaesth..

[41] Watanabe H., Nakazawa H., Tokumine J., Yorozu T. (2025). Real-time vs. static ultrasound-guided needle cricothyroidotomy: A randomized crossover simulation trial. Sci. Rep..

[42] Dotson M., Thompson S., Singh G.P., Area S. (2024). Comparison of Point-of-Care Ultrasound Guidance and the Traditional Approach in Performing Simulated Emergent Cricothyroidotomy Among Emergency Medicine Physicians, Residents, and Students. Cureus.

[43] Siddiqui N., Yu E., Boulis S., You-Ten K.E. (2018). Ultrasound Is Superior to Palpation in Identifying the Cricothyroid Membrane in Subjects with Poorly Defined Neck Landmarks: A Randomized Clinical Trial. Anesthesiology.

[44] Nakazawa H., Uzawa K., Tokumine J., Lefor A.K., Motoyasu A., Yorozu T. (2023). Airway ultrasound for patients anticipated to have a difficult airway: Perspective for personalized medicine. World J. Clin. Cases.

[45] Adi O., Fong C.P., Sum K.M., Ahmad A.H. (2020). Usage of airway ultrasound as an assessment and prediction tool of a difficult airway management. Am. J. Emerg. Med..

